# How do we define therapy-resistant constipation in children aged 4–18 years old? A systematic review with meta-narrative synthesis

**DOI:** 10.1136/bmjpo-2023-002380

**Published:** 2024-06-06

**Authors:** Vassiliki Sinopoulou, Morris Gordon, Shaman Rajindrajith, Watshala Hathagoda, Aditi Bhupendra Rane, Anita Sedghi, Merit Tabbers, Carlo Di Lorenzo, Miguel Saps, Marc A Benninga

**Affiliations:** 1 University of Central Lancashire, Preston, UK; 2 University of Colombo, Colombo, Sri Lanka; 3 University of Amsterdam, Amsterdam, The Netherlands; 4 Division of Gastroenterology, Hepatology and Nutrition, Nationwide Children's Hospital, Columbus, Ohio, USA; 5 University of Miami Miller School of Medicine, Miami, Florida, USA; 6 Department of Pediatrics, Emma Childrens' Hospital UMC, Amsterdam, The Netherlands

**Keywords:** gastroenterology, qualitative research

## Abstract

**Background:**

Therapy-resistant constipation often is a frustrating clinical entity recognised by the persistence of infrequent and painful bowel movements faecal incontinence and abdominal pain despite intensive treatment. It is important to clearly define therapy-resistant constipation before children are subjected to invasive diagnostic and therapeutic procedures.

**Aim:**

To conduct a systematic review determining how paediatric interventional studies define therapy-resistant constipation.

**Method:**

We searched CENTRAL, MEDLINE, Embase, WHO ICTR and ClinicalTrials.gov. Studies that included patients with therapy-resistant constipation were identified. Data were extracted on criteria used for defining therapy-resistant constipation and reported using a meta-narrative approach highlighting areas of convergence and divergence in the findings.

**Results:**

A total of 1553 abstracts were screened in duplicate, and 47 studies were included in the review. There were at least seven definitions used in the paediatric literature to define medically resistant constipation. The term intractable was used in 24 articles and 21 used the term refractory to describe therapy-resistant constipation. Out of them, only 14 articles have attempted to provide an explicit definition including a predefined time and prior therapy. There were 10 studies without a clear definition for therapy-resistant constipation. The duration before being diagnosed as therapy-resistant constipation varied from 1 months to 2 years among studies. Seven studies employed the Rome criteria (Rome III or Rome IV) to characterising constipation while five adopted the Rome III and European and North American paediatric societies definition of paediatric gastroenterology, hepatology and nutrition guideline of management of constipation in children.

**Conclusion:**

The current literature has no explicit definition for therapy-resistant constipation in children. There is a need for a detailed consensus definition to ensure consistency of future research and to avoid unnecessary and maybe even harmful, invasive diagnostic and therapeutic interventions.

**WHAT IS ALREADY KNOWN ON THIS TOPIC**

- Childhood therapy-resistant constipation is a common and painful condition, often managed with invasive therapies. However, a consensus definition and diagnosis does not exist.

**WHAT THIS STUDY ADDS**

- The existing literature for interventions on therapy-resistant constipation often does not define it. When it does there is inconsistency around duration of symptoms and previous therapy failure.

**HOW THIS STUDY MIGHT AFFECT RESEARCH, PRACTICE OR POLICY**

- This review can lead to a consensus definition and diagnostic criteria for therapy-resistant constipation. In turn, this will aid appropriate management and consistency in future research.

## Introduction

Functional constipation is a common gastrointestinal disorder that affects children globally. Based on the available data, it has a pooled prevalence of 9.5%.^1^ Constipation is a frequent cause of emergency department visits.^2^ It can result in substantial use of clinical resources in outpatient departments, particularly among children.^3^ Additionally, constipation can significantly impact public funds through annual health budgets, directly and indirectly.^4^

Several guidelines describe the management of childhood constipation.^5–7^ Even with optimal management, about one-third of children are deemed to have therapy-resistant constipation.^8^ Although childhood functional constipation is clearly defined using the Rome criteria, there is no such definition for children not responding to optimal management.^9^ Several authors have defined therapy-resistant constipation using the duration of unresponsiveness to medical management varying from 3 months to 2 years without clear consensus.^5 10^ In addition, clinicians and researchers use the terms ‘refractory’ and ‘intractable’ interchangeably, complicating the definition of therapy-resistant constipation.

Therapy-resistant constipation has long-term physical and psychological complications.^11–13^ In addition, some children with therapy-resistant constipation undergo invasive diagnostic tests such as barium enema, defecography, anorectal and/or colorectal manometry. The majority of these children need (a combination of) oral laxatives, enemas or transanal irrigation. A smaller proportion requires even needs surgical interventions, such as sacral neuromodulation, antegrade continence enema, the formation of diversion stomas and surgical resection of the bowel, or subtotal colectomy, with ileorectal anastomosis, all interventions which have significant morbidity and a high incidence of complications.^14^ Therefore, it is imperative to clearly define therapy-resistant constipation to ensure consistent deployment of therapies to this group and consistent understanding of the goals and outcomes of therapies in these circumstances. Against this backdrop, we aimed to conduct a systematic review to determine how interventional studies define the condition and propose a way forward for an internationally accepted definition.

## Methods

A plan for this systematic review was prospectively registered in PROSPERO (CRD42022371846).

### Literature search

A literature search was conducted using CENTRAL, MEDLINE, Embase, WHO ICTR and ClinicalTrials.gov and searched for studies meeting the inclusion criteria. Our search strategy was “(Intractable OR Refractory OR Non-respons*) AND constipation* AND child*)”. The age limit was set from 2 to 18 years, and the search was performed in November 2022.

The included studies reference of all Cochrane systematic reviews for constipation in childhood were also handsearched. We followed the Preferred Reporting Items for Systematic Reviews and Meta-Analyses 2020 checklist.

### Inclusion and exclusion criteria

#### Inclusion criteria

All published papers from January 1995 to October 2022, on intractable/refractory constipation, in correspondence with the release of the Consolidated Standards of Reporting Trials statement to the current date, were included.

Type of participants: Patients with therapy-resistant constipation, between 4 and 18 years of age.

Types of interventions: Studies that included and compared any form of intervention and dosage of drugs or no intervention.

Types of outcomes: Any outcome measures.

#### Exclusion criteria

Studies on adults and children younger than 4 years, non-intractable/refractory constipation, articles written in non-English languages, opinion pieces, commentaries, editorials, secondary evidence and review articles and non-interventional works.

### Data collection and analysis

#### Title/abstract and full-text screening

All potential studies were reviewed independently by two authors (ABR and WH) for title/abstract screening, followed by full-text screening (ABR, WH and AS). Any conflicts were resolved by a fourth author (MG and VS).

#### Data extraction

All included studies underwent data extraction independently by three authors (ABR, WH and AS), and disagreements were resolved by a fourth author (MG and VS).

Data were extracted based on the following headings:

- Definitions for therapy-resistant constipation (or descriptions that can be characterised as definitions).
- Reference for the definition (if given).
- Classification of definition as explicit or implicit.
- Inclusion criteria for each study.
- Exclusion criteria for each study.
- Type of study (randomised clinical trial, non-randomised clinical trial, cross-sectional, etc).
- Age of included children.
- Country(ies) of study origin.

All the included studies’ characteristics were manually collected and recorded within a database file.

#### Definition of refractory/intractable constipation

We classified studies based on the type of definition: explicit or implicit. Then identified the most common themes within the definitions.

- Mention of a time frame in the definition.
- Mention of bowel frequency.
- Use of Rome criteria.
- Reference.
- Interactable/refractory or both.
- Previous therapy.

Data were reported using a meta-narrative approach, highlighting areas of convergence and divergence in the findings. Meta-narrative review is a relatively new method of systematic review, designed for topics that have been differently conceptualised and studied by different groups of researchers. We followed the RAMESES publication standards for meta-narrative reviews.^15^

#### Data analysis

All the categorical data were presented as tables and figures, and no numerical data were included in the analysis.

#### Risk of bias analysis

No risk of bias analysis applicable.

The level of bias of the included studies does not affect the definition of intractable constipation, which is the only outcome of interest of this meta-narrative review.

#### Patient and public involvement

No patients were involved in this review.

## Results

**Figure 1:**
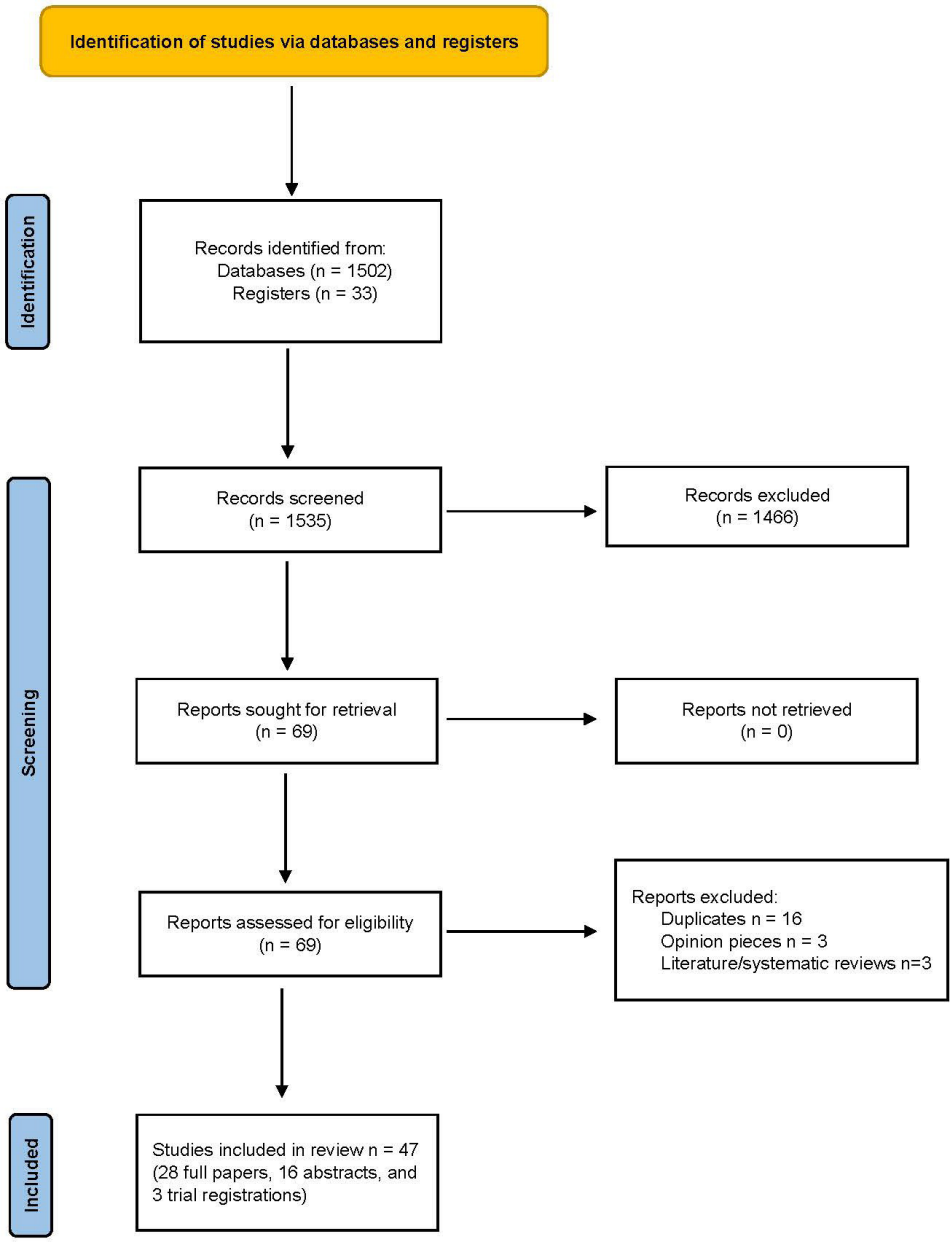
PRISMA flow chart. PRISMA, Preferred Reporting Items for Systematic Reviews and Meta-Analyses.

A total of 1535 studies were identified on a search conducted on the 3 November 2022, 1466, of which were excluded as they did not meet the inclusion criteria for this review. 69 studies were screened for eligibility. 25 studies were excluded: duplicates (16), opinion pieces (3) and literature/systematic reviews (3). A total of 47 studies (28 full papers, 16 abstracts and 3 trial registrations) were included and downloaded for data extraction as PDF files (figure 1).

### Description and characteristics of included studies

The 47 studies included in the systematic review comprised 28 full papers, 3 clinical trial registrations and 16 abstracts. The year of publication of the studies was from 1996 to 2022. The studies came from diverse geographical regions; from North America, Europe, Asia, Australia, South America. Online supplemental table 1 provides study characteristics including design, definition of therapy-resistant constipation used by the researchers, terminology used to define therapy resistant constipation, duration of treatment before being diagnosed with therapy-resistant constipation and the source of reference.

10.1136/bmjpo-2023-002380.supp1 Supplementary data

### Definitions of therapy-resistant constipation

There were at least seven clear definitions of therapy- resistant constipation for children in the published literature included in this review. Most of these variations are due to the duration of therapeutic interventions before labelling children as having therapy-resistant constipation and the terminology used to define therapy-resistant constipation. Some of them included refractory constipation: symptoms not responsive to conventional therapy,^16^ functional constipation unresponsive to optimal conventional treatment for at least 3 months,^17^ chronic constipation not responding to maximum laxative therapy, behavioural therapy and toilet-training programme with duration of symptoms of >2 years,^18^ and all children presenting with chronic constipation and showing no response to rigorous medical management over a period of 1 month or more.^19^ It is important to note that 10 articles had no clear definition at all.

### Terminology (refractory or intractable)

21 studies^16 19–37^ used the word refractory while 24 studies^10 17 18 38–58^ used the word intractable to describe treatment resistant constipation. Two studies used refractory and intractable interchangeably.^59 60^

### Studies with an explicit definition

**Figure 2:**
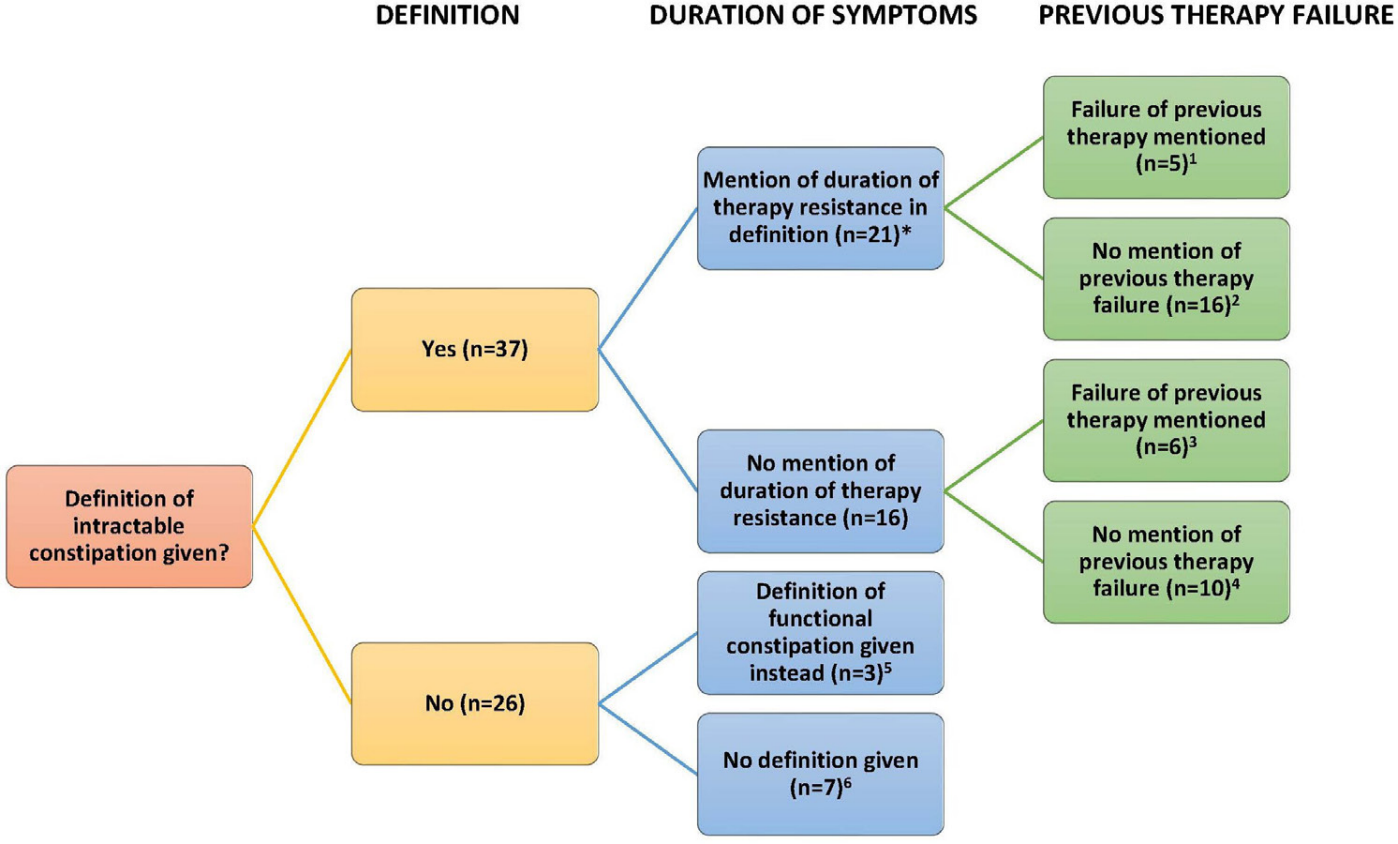
Schematic representation of the contents of the definitions or other related details, for therapy-resistant constipation by the included studies’. ^1^Yik *et al*,^18^ Bellomo-Brandao *et al*,^21^ Arruda *et al*,^59^ Noviello *et al*,^60^ Mousavi *et al*.^47^ ^2^Hynes *et al*,^53^ Rawat *et al*,^48^ RBR-7mry33, Kajbafzadeh *et al*,^43^ Ng *et al*,^42^ Gupta *et al*,^39^ Puoti *et al*,^25^ Monjaraz *et al*,^52^ Kuizenga-Wessel *et al*,^40^ Koppen *et al*,^51^ Wessel *et al*,^44^ Campos *et al*,^24^ Nurko *et al*,^10^ RBR-344jq8, Redkar *et al*,^19^ Redkar *et al*.^36^ ^3^Bonilla *et al*,^16^ Heitmann *et al*,^23^ Gomez-Suarez *et al*,^37^ van Wunnik *et al*,^35^ Haddad *et al*,^46^ Gonzalez *et al*.^28^ ^4^Baalem *et al*,^41^ Omar *et al*,^22^ Arbizu *et al*,^26^ van der Wilt *et al*,^27^ van der Wilt *et al*,^61^ Christison Lagay *et al*,^38^ Levitt *et al*,^45^ Tang *et al*,^20^ Youssef *et al*,^54^ IRCT20111229008554N4. ^5^Koppen *et al* 17 (Rome III criteria), Vriesman *et al* 56 (Rome IV criteria), Motion *et al* (NICE guidelines). ^6^Menakaya *et al*,^55^ Zacur *et al*,^31^ Carr *et al*,^30^ Koppen *et al*,^17^ van der Wilt *et al*,^27^ Bellomo-Brandao *et al*,^50^ Valitutti *et al*.^29^ *At least 2 years: Yik *et al*,^18^ Hynes *et al*;^53^ at least 12 months: Bellomo-Brandao *et al*,^21^ Arruda *et al*,^59^ Rawat *et al*,^48^ RBR-7mry33; at least 6 months: Kajbafzad *et al*;^43^ at least 3 months: Noviello *et al*,^60^ Gupta *et al*,^39^ Puoti *et al*,^25^ Monjaraz *et al*,^52^ Kuizenga-Wessel *et al*,^40^ Koppen *et al*,^17^ Wessel *et al*,^44^ Mousavi *et al*,^47^ Campos *et al*,^24^ Nurko *et al*,^10^ RBR-344jq8; at least 1 month: Redkar *et al*,^19^ Redkar *et al*.^36^

Out of 47 studies, 14 studies provided an explicit definition for therapy-resistant constipation. The definitions combined varying components such as duration of treatment, specifications of therapy and nature of stools. Among them, duration of medical treatment was the most frequently used component in defining therapy-resistant constipation. Of the 47 studies which included duration of treatment as part of the definition, 2 studies considered no responsiveness of 2 years or more to treatment,^18 53^ 3 considered unresponsiveness to treatment more than 12 months,^21 48 59^ 2 studies considered no responsiveness of 6 months to treatment,^42 43^ 10 studies used duration of treatment more than 3 months^10 17 24 25 39 40 44 47 52 60^ and 1 study defined therapy-resistant constipation as having bowel movement less than 3 per week for at least 2 months prior to diagnosis^36^ (figure 2).

A total of 15 studies defined therapy-resistant constipation without reporting treatment duration.^16 20 22 23 26–28 35 37 38 41 45 46 54 61^

### Studies with no structured definition

Seven studies used the Rome criteria (in general, even though the Rome criteria does not define it) to define therapy-resistant constipation. Among them, one study used the Rome IV criteria without s specific duration of treatment.^22^ Five studies used Rome III criteria with a definitive duration of treatment.^24 42 43 59 61^ One RCT (Ranodmised controlled trial) defined therapy-resistant constipation fulfilling the Rome IV criteria for three or more months.^49^ 10 studies did not provide a clear definition for therapy-resistant constipation^17 29–32 50 55–58^ (figure 2).

## Discussion

Therapy-resistant constipation is a common and formidable challenge in paediatric clinical practice. It is crucial to have a clear and explicit definition of this clinical entity in order to implement appropriate management strategies at an early stage that may improve outcomes. The Rome criteria clearly define functional constipation in infants, toddlers and children.^9 62^ However, after an extensive review of the existing paediatric literature, we were unable to find a clear definition for therapy-resistant constipation for children, especially in terms of the duration of unresponsiveness to optimal medial management before being labelled as therapy-resistant constipation. Although the National Institute for Clinical Excellence (NICE, UK) has defined therapy-resistant constipation, the duration of symptoms of constipation is, however, not included in their definition.^7^ Widely varying definitions found in our review show the lack of consensus among these definitions. We believe that it is imperative to use unambiguous terminology that includes, rigorous criteria of failure, type of therapeutic interventions and their precise duration in defining therapy-resistant constipation.

### Terminology of therapy-resistant constipation

Several studies have used the term intractable^10 17 18 38–58^ while others have used the term refractory.^16 19–37^ It is interesting to note that some studies have used both terms.^59 60^ Although, it is clear that both terms are being used in the definition of medically unresponsive constipation, the literature shows no agreement on the terminology and use the terms refractory and intractable loosely and interchangeably. It is important for researchers and healthcare professionals to come to a consensus on the terminology used to describe therapy-resistant constipation as it helps to understand the pathophysiology, recognise symptomatology, use the correct diagnostic tools, compare treatment regimens and design clinical trials.

### Time frame of therapy-resistant constipation

It is also evident that there is no clear agreement among studies on the duration of medical treatment before children are deemed to be considered as therapy-resistant constipation. Among the studies that provided an explicit definition for therapy-resistant constipation, there is no definitive time duration that can be used as a benchmark. Most studies with an explicit definition seem to believe that symptoms must persist for at least 3 months to meet the criteria for therapy-resistant constipation^10 17 39 40 52 60^ while some studies have set a longer time frame of 12 (4/47)^21 33 48 59^ or 24 months (2/47),^18 53^ respectively. Among those studies that do offer an explicit definition, there is still no consensus about how long symptoms need to persist in order to be considered as therapy resistant.

### Studies with no clear definition for therapy-resistant constipation

We also found that a significant number of studies have not attempted to clearly define medically unresponsive constipation.^29–32 50 51 55–58^ In those studies, there was no clear identification of duration of medical unresponsiveness. Although beyond the scope of defining the therapy-resistant constipation, some studies which had not clearly defined the unresponsiveness have reported outcomes of major surgical interventions as treatments for children. We believe this is one of the reasons that demands an internationally accepted definition for medically unresponsive constipation in children. Other reasons why we need a standard definition include, harmonising research in this important disease entity and identifying epidemiological and pathophysiological nuances related to refractory/intractable constipation.

### Studies that used the variations of Rome criteria to define therapy-resistant constipation

The Rome criteria do not provide a clear definition for therapy-resistant constipation. However, we found that a notable proportion of studies (10 out of 47) have used the Rome criteria to describe refractory constipation. One study implemented the Rome IV criteria without specifying a duration,^22^ four studies employed the Rome III/IV criteria with a specific duration.^34 35 38 61^ Three studies employed Rome III criteria^42 43 59^ and one study used Rome IV criteria^49^ with specific duration. Among the studies that established a duration, some considered a period of 6 months or longer,^42 43^ one study used a period of 12 months^59^ while others required the fulfilment of the Rome III/IV criteria for a minimum of 3 months of treatment.^24 49^ It is evident that researchers look on the Rome process to have a definition for medically unresponsive constipation. This reinforces the importance of having a clear and consistent definition for medically refractory constipation in future iterations of the Rome criteria to ensure the high quality and validity of research findings on childhood constipation as well as optimal care for those with severe unresponsive constipation.

### Studies with description with prior medical therapy

11 studies with an explicit definition have considered prior medical therapy before being considered as therapy-resistant constipation. These therapies include maximum doses of osmotic and stimulant laxatives, and extensive behavioural therapy and toilet training.^16 18 21 23 28 35 37 46 47 59 60^ All three guidelines published by NICE, ESPGHAN/NASPGHAN, and Indian Academy of Paediatrics have defined the standard management.^5–7^ High-dose polyethylene glycol is used to evacuate the rectal faecal mass as the first step and rectal enemas and suppositories are used when there is poor response to polyethylene glycol. All three guidelines agreed on polyethylene glycol-based therapy as the first-line maintenance therapy for childhood constipation and stimulant laxatives are added when there is a poor response. Therefore, we believe that there should be consensus on the choice of drugs, their dosages, the order of usage of different laxatives, both oral and rectal laxatives and the duration of therapy. The recommended therapy in these guidelines can be used as a steppingstone in defining the optimal medical intervention before being labelled as therapy-resistant constipation.

### Previous literature on defining therapy-resistant constipation

A previous systematic review analysing adult literature has also attempted to define pharmacologically therapy-resistant constipation in adults. In this study, 61 papers were reviewed to define pharmacologically therapy-resistant constipation. Similar to our findings, they also found the terminology of severe, refractory and intractable interchanging being used without consensus. In addition, the duration of therapy for constipation prior to be labelled as therapy resistant varied from 6 to 12 months with some studies not specifying the duration but only mentioning several years.^63^

### Strengths and limitations

This review has several strengths. We searched a number of databases to identify the relevant literature and included all possible papers as well as abstracts which had defined therapy-resistant constipation. We identified articles which had both explicit as well as implicit definitions, therefore, were able to understand the components that are needed to scientifically define therapy-resistant constipation. It was decided not to restrict inclusion of the articles based on the quality assessment as that had no implication on the definition of therapy-resistant constipation. A limitation of our study is that we excluded articles published in non-English languages where we could have missed some of the definitions. However, observing the trends of definitions used in articles published in the English language, it is unlikely that this would affect the overall conclusions of the present article.

It is important to establish consensus on a definition for this clinical paradigm. As many aspects of the different definitions given in the literature directly inform the choice of therapeutic goals for patients, professionals and researchers, clarity on these definitions will directly inform such practice. It is possible that this research has uncovered a spectrum of overlapping but distinct clinical presentations. It is also possible that a single consensus is needed with other incomplete understandings of this clinical phenomenon as identified in our findings, rejected by the clinical community. It is, therefore, vital as a future and relatively urgent research goal to reach such an international consensus. The most appropriate method to achieve this would be through either a round table or Delphi process.

### Conclusions

We conclude by stating although there is a significant literature on therapy-resistant constipation in children; however, there is no consensus definition in terms of the terminology, the maximum medical treatment and duration of maximum medical intervention before identifying as having medically unresponsive constipation. It is crucial to clearly define therapy-resistant constipation in children as it significantly impacts the management and outcome and can prevent unnecessary and potentially harmful further investigations and invasive treatment. It is important to establish a consensus and incorporate this definition into guidelines and criteria to ensure consistency in treatment.

## Data Availability

Data are available on reasonable request. Data will be provided on reasonable request from the contact author.
