# Supplementary material for: How do we define therapy-resistant constipation in children aged 4–18 years old? A systematic review with meta-narrative synthesis
Source: BMJ Paediatr Open. 2024 Jun 6;8(1):e002380. doi: 10.1136/bmjpo-2023-002380 (PMC11163682; doi:10.1136/bmjpo-2023-002380)
Supplement: Supplementary data [file bmjpo-2023-002380supp001.pdf]

Table 1: Studies included in the reviews

	Study ID	Study design	Definition	Duration before diagnosing treatment resistant constipation	Intractable/refractory/ or both	Source of reference
**Studies with an explicit definition**						
1	Bonilla 2020	retrospective review	Refractory constipation: symptoms not responsive to conventional therapy.	Not mentioned	Refractory	NR
2	Gupta 2020	Retrospective review	'Intractable constipation' (IC) was defined as that unresponsive to optimal conven- tional treatment for at least 3 months.	3 months	Intractable	NR
3	Noviello 2020	Retrospective study	Chronic refractory constipation or intractable constipation: children with a need for daily rectal stimulation (enemas or suppositories) despite laxatives for more than 3 months.	3 months	Both	NR
4	Bellomo-Brandao 2018	Retrospective study	ORSI persistence after 12 month-follow-up periods under conservative therapy (dietary fiber, oral	12 months	Refractory	NR

			osmotic laxatives, and rectal enemas).			
5	Monjaraz 2018	Retrospective cohort study	no response from treatment with usual doses of laxative for more than three months	3 months	Intractable	NR
6	Koppen 2017	Cross sectional survey	Functional constipation unresponsive to optimal conventional treatment for at least 3 months is referred to as intractable FC. In children with intractable FC, surgery is sometimes required. Conventional treatment of FC in children comprises of education, a toilet training program, and pharmacological treatment with oral and/or rectal laxatives	3 months	Intractable	ESPGHAN/NASPGHAN guideline 2014
7	Kuizenga-Wessel 2017	Retrospective chart study	failure to respond to intensive conventional therapy for ≥3 months as described in the ESPGHAN-NASPGHAN guidelines for FC	3 months	Intractable	ESPGHAN-NASPGHAN guidelines 2014
8	van Wunnik 2012	Retrospective review	functional constipation (once organic causes	Not mentioned	Refractory	Rome III

			such as anatomical or neurological abnormalities and endocrine or metabolic disorders are ruled out, and when they meet the ROME III criteria: the presence of at not responding to intensive oral and rectal laxative treatment			
9	Yik 2011	Retrospective review	Defined as chronic constipation not responding to maximal laxative therapy, behavioral therapy, and toilet-training program with duration of symptoms of > 2 years.<br><br>Medical treatment–resistant group	2 years	Intractable	NR

10	Christison-Lagay 2010	Retrospective review	Intractable constipation, defined as failure to respond to conventional therapy as described by the North American Society of Pediatric Gastroenterology and Nutrition (1,2). Children younger than 4 years (n = 5), who cannot strictly meet Rome III criteria, had encopresis and 2 or fewer defecations per week.	Not mentioned	Intractable	NASPGHAN guideline 1999<br><br>Rome III guideline 2006
11	Tang 2009	single-center retrospective review	Refractory constipation was defined as failing conventional therapy with <3 stools per week and poor satisfaction of response from patient/parent assessment	Not mentioned	Refractory	NR
12	Nurko 1996	open trial/ non-randomised intervention	constipation was diagnosed if the child had less than three stools per week); intractable constipation was defined as long-standing constipation (more than 6 months in duration), with no	12 weeks	Intractable	NR

			response after at least 12 weeks of intensive medical therapy			
13	RBR-7mry33	Non-randomized-controlled trial	refractory constipation criteria that is: persistence of retentive incontinence for at least 12 months on maximum tolerable doses of oral laxatives and rectal enemas	12 months	Refractory	NR
14	IRCT20111229008554N4 (1)	RCT	Refractory constipation according to Rome III criteria	Not mentioned	Refractory	Rome III
**Studies with an implicit definition**						
15	Baaleman 2022	pilot prospective cohort study	constipation refractory to conventional treatment	Not mentioned	Intractable	NR
16	Campos 2021	Retrospective cohort study	no response to optimal conventional treatment for at least 3 months.	3 months	Refractory	The definition of RC is referenced in the study, with the criteria being attributed to ESPGHAN and NASPGHAN.
17	Omar 2021	NR	The Rome IV diagnostic criteria was used to define patients with refractory constipation.	Not mentioned	Refractory	Rome IV

18	Arruda 2020	interventional study	When the therapeutic response is unsatisfactory at maximum doses of laxatives associated with the use of enemas and there are no alarm signals to investigate an organic cause justifying the symptoms, the condition is scarce. termed refractory or intractable intestinal constipation. The diagnosis of RFC was established when RFI persisted after at least 12 months of follow-up with the medical specialty, during treatment with maximum tolerable doses of osmotic laxatives, and rectal enemas	12 months	Both	ESPGHAN/NASPGHAN guideline 2-14 and Rome III criteria. 1.
19	Heitmann 2020	Retrospective review	constipation refractory to intensive medical treatment, included a variable combination of per oral osmotic and stimulant laxatives including one, or a combination of, the following; bisacodyl, polyethylene glycol with	Not mentioned	Refractory	NR

			electrolytes, so- dium picosulfate, prucalopride, lubiprostone, senna, lactulose, mag- nesium oxide, magnesium citrate. Per rectal enema and/or rectal irrigation was additionally administered for some children.			
20	Kajbafzadeh 2020	Randomised clinical trial	Intractable functional constipation based on Rome III criteria and paraclinical tests (i.e., measurement of rectum diameter by ultrasound and anorectal manometry), defecation frequency of less than 3 times per week, positive history for passing of hard stool, episodes of fecal soiling, abnormal stool form (Bristol Stool Form 1–3) for 6 months	6 months	Intractable	Rome III
21	Puoti 2019	retrospective cohort study	failure to respond to optimum conventional therapy for at least 3 months	3 months	Refractory	NR
22	Redkar 2018	Retrospective study	no response to rigorous medical management over a period of 1	1 month	Refractory	NR

			month or more			
23	Arbizu 2017	single center prospective study	patients that have previously failed conventional medical therapy	Not mentioned	Refractory	NR
24	Hynes 2017	Retrospective review	symptoms for >2 years not responding to treatment	2 years	Intractable	NR
25	Gomez-Suarez 2016	exploratory retrospective case series	no definition. (Conventional medical management of constipation involves a combination of dietary, behavioral, and medical management.. When conventional medical therapy is ineffective, surgical management is often necessary.	Not mentioned	Refractory	NR
26	Wessel 2016	Non-randomised intervention	When symptoms are irresponsive to optimal conventional treatment for at least 3 months, this is referred to as intractable constipation.	3 months	Intractable	evidence-based recommendations from ESPGHAN and NASP- GHAN. J Pediatr Gastroenterol Nutr 2014; 58: 258–74.
27	van der Wilt 2016	prospective cohort study	No definition. (Functional constipation, as defined by the ROME III criteria. chronic (>1 year) constipation refractory to	Not mentioned	Refractory	NR

			conservative treatment, were eligible to be included in this study.			
28	Mousavi 2014	prospective cohort study	intractable constipation that did not respond to classic conservative treatment (diet, laxatives or enema) over a period of 3 months or more	3 months	Intractable	NR
29	Ng 2014	Retospective study	1. Anal sphincter dysynergia is more commonly seen in those with sphincter hypertonicity, which may imply that both external and internal anal sphincters contribute to the pathophysiology of intractable constipation.<br><br>2. (i) chronic constipation according to the Rome III criteria, or (ii) complications due to fecal retention needing hospitalisation, followed by poor response to a 6-month period of conservative treatment.	6 months	Intractable	Rome III
30	Van Der Wilt 2014	A prospective cohort study	Patients with constipation refractory	Not mentioned	Refractory	Rome III

		(non randomized intervention)	to conservative treatment according to the Rome III			
31	Redkar 2012	Retrospective study	All children presenting with chronic constipation and showing no response to rigorous medical management over a period of 1 month or more	1 month	Refractory	NR
32	Gonzalez 2011	Case series	Lactulose, stimulants such as bisacodyl or sennoside, poly-ethylene glycol, and rectal enemas are the treatments most frequently used in constipation in critically ill children and adults. Occasionally, however, the condition does not respond to these drugs, and this can give rise to cases of therapy-resistant constipation for which other therapeutic measures are needed.	Not mentioned	Refractory	NR
33	Levitt 2009	Case series	intractable severe constipation with soiling (encopresis) that is unresponsive to standard medical	Not mentioned	Intractable	NR

			therapy, have refractory symptoms,			
34	Rawat 2004	non randomised intervention	Although most children with chronic constipation respond to conventional therapies, a small proportion have intractable constipation refractory to nonoperative treatment modalities. All children were fecally incontinent (soiling at least once per day) and had undergone a variety of conventional treatments over a median of 4.4 years (range 1.2-10 years). All had optimal medical therapy under the care of pediatric gastroenterologists for a minimum of 1 year.	12 months	Intractable	NR
35	Haddad 2002	non-randomised inervention	They all had intractable constipation with daily soiling. All were fecally incontinent, and all had had manual evacuations under general anesthetic. Failed medical therapy needing variety of treatments over a 3- to 13-year period, ranging from stimulant and bulk laxatives to	Not mentioned	Intractable	NR

			suppositories and enemas, with all failing to respond.			
36	Youssef 2002	Retrospective review	Constipation in children who are neurologically normal and who had failed conventional medical treatments for constipation.	Not mentioned	Intractable	NR
37	RBR-344jq8	RCT	diagnosis of CIC, established according to the criteria of Rome IV, and classified as having intractable intestinal constipation, according to ESPGHAN/NASPGHAN guideline (after a period of three months)	3 months	Intractable	Rome IV and ESPGHAN/NASPGHAN guideline
**Studies with no definition**						
38	Menakaya 2022	Retrospective Study	NR	Not mentioned	Intractable	NR
39	Motion 2022	non-randomised Intervention	NR	Not mentioned	Refractory	NR
40	Zacur 2021	cross-sectional observational study	NR	Not mentioned	Refractory	NR
41	Vriesman 2018	retrospective cohort study	NR	Not mentioned	Intractable	NR

42	Carr 2017	retrospective study	NR	Not mentioned	Refractory	NR
43	koppen 2017 (2)	retrospective study	NR	Not mentioned	Intractable	NR
44	Koppen 2017 (4)	retrospective study	NR	Not mentioned	Intractable	NR
45	van der Wilt 2017	Retrospective review	NR	Not mentioned	Refractory	NR
46	Valitutti 2016	retrospective analysis of case series	NR	Not mentioned	Intractable	NR
47	Bellomo-Brandao 2003	A double-blind, placebo controlled, crossover study	NR	Not mentioned	Intractable	NR
